# Shoulder Proprioception and Its Correlation with Pain Intensity and Functional Disability in Individuals with Subacromial Impingement Syndrome—A Cross-Sectional Study

**DOI:** 10.3390/diagnostics13122099

**Published:** 2023-06-17

**Authors:** Fareed F. Alfaya, Ravi Shankar Reddy, Batool Abdulelah Alkhamis, Praveen Kumar Kandakurti, Debjani Mukherjee

**Affiliations:** 1 Department of Orthopaedic Surgery, College of Medicine, King Khalid University, Abha 61421, Saudi Arabia; ffalfaia@kku.edu.sa; 2 Department of Medical Rehabilitation Sciences, College of Applied Medical Sciences, King Khalid University, Abha 61421, Saudi Arabia; balkamees@kku.edu.sa (B.A.A.); debjani@kku.edu.sa (D.M.); 3 College of Health Sciences, Gulf Medical University, Ajman 4184, United Arab Emirates; dean.coahs@gmu.ac.ae

**Keywords:** shoulder proprioception, subacromial impingement syndrome, pain intensity, functional disability

## Abstract

Subacromial Impingement Syndrome (SAIS) is a common shoulder condition characterized by pain and functional impairment. Proprioception, the sense of joint position and movement, is crucial in maintaining joint stability and coordinating movements. The relationship between shoulder proprioception, pain intensity, and functional disability in individuals with SAIS remains unclear, with conflicting findings in the literature. This cross-sectional study aimed to evaluate shoulder proprioception, examine its correlation with pain intensity and functional disability, and contribute to our understanding of the clinical implications of proprioceptive deficits in individuals with SAIS. Forty-two individuals were diagnosed with SAIS, and an equal number of asymptomatic controls were recruited. Shoulder proprioception was assessed using a digital inclinometer, measuring joint position sense at various angles of flexion and rotation. Pain intensity was measured using the Visual Analog Scale (VAS), and functional disability was assessed using the Shoulder Pain and Disability Index (SPADI). Results: Individuals with SAIS exhibited significantly higher joint position error (JPE) values compared to asymptomatic controls in all measured angles of flexion and rotation (*p* < 0.001). Strong positive correlations were observed between JPE and pain intensity (r = 0.61 to 0.71, *p* < 0.01) and disability (r = 0.56 to 0.68, *p* < 0.01). These findings suggest impaired shoulder proprioception is associated with higher pain intensity and functional disability in SAIS. This study provides evidence of impaired shoulder proprioception in individuals with SAIS and its correlation with pain intensity and functional disability. The results highlight the clinical relevance of proprioceptive deficits in SAIS and emphasize the importance of incorporating proprioceptive assessment and targeted rehabilitation interventions into managing this condition. Future research should focus on longitudinal studies with larger and more diverse samples to further understand the underlying mechanisms and evaluate the effectiveness of proprioceptive interventions in improving outcomes for individuals with SAIS.

## 1. Introduction

Subacromial Impingement Syndrome (SAIS) is a common shoulder condition characterized by pain and functional impairment, primarily affecting the rotator cuff tendons and subacromial bursa [1]. It is associated with various etiological factors, including repetitive overhead activities, shoulder instability, and anatomical variations [2]. One significant aspect of SAIS that has gained attention in recent research is shoulder proprioception, which plays a crucial role in maintaining joint stability and coordinating movements [3].

Proprioception refers to the sense of joint position and movement in space, which allows individuals to perceive and control their limb’s position without relying solely on visual feedback [4]. Shoulder proprioception is vital in various daily activities, including reaching, grasping, and performing precise movements [5]. Any disruption in shoulder proprioception can lead to altered motor control, compromised joint stability, and functional limitations [6].

Understanding the relationship between shoulder proprioception, pain intensity, and functional disability in individuals with SAIS is essential for effective management and rehabilitation strategies. Previous studies have reported conflicting findings regarding proprioceptive deficits in SAIS patients [7,8,9]. Some studies have suggested impaired proprioception, while others have not found significant differences when compared to healthy individuals [7,8]. Furthermore, the correlation between shoulder proprioception and pain intensity or functional disability in SAIS remains unclear. For instance, a study by Haik et al. [10] examined proprioceptive acuity in individuals with SAIS and found no significant differences compared to a healthy control group. Similarly, a study by Gomes et al. [11] reported no differences in proprioception between individuals with SAIS and asymptomatic controls. These findings suggest that impaired proprioception may not be a consistent feature of SAIS. On the other hand, several studies have demonstrated proprioceptive deficits in individuals with SAIS [9,12]. Sahin et al. [13] investigated shoulder proprioception in patients with SAIS and found significant impairments compared to a control group. Similarly, a study by Keenan et al. [12] reported decreased shoulder joint position sense in individuals with SAIS. These studies suggest that impaired proprioception may contribute to the pathophysiology of SAIS.

Furthermore, the correlation between shoulder proprioception and pain intensity or functional disability in SAIS remains unclear. The relationship between proprioception and pain intensity or functional disability has been extensively investigated in various joints, but its exploration, specifically in the shoulder, remains limited [14,15,16]. Some studies have reported positive correlations, indicating that individuals with poorer proprioception also experience higher levels of pain intensity and functional disability [14,15,16]. Clarifying these relationships could provide valuable insights into the underlying mechanisms of SAIS and inform the development of targeted management and rehabilitation strategies.

To assess pain intensity, we utilized the Visual Analog Scale (VAS), a well-established self-report measure widely used in clinical and research settings [17]. The VAS allows individuals to rate their pain continuously, quantifying its severity [18]. On the other hand, functional disability was assessed using the Shoulder Pain and Disability Index (SPADI), a validated questionnaire specifically designed to evaluate shoulder-related functional limitations and disability [19]. The SPADI encompasses subscales for pain and disability, offering a comprehensive assessment of the impact of SAIS on individuals’ daily activities and quality of life [19].

By investigating the correlation between shoulder proprioception, pain intensity, and functional disability in individuals diagnosed with SAIS, we aim to contribute to the existing body of knowledge regarding the proprioceptive impairments associated with this condition. The findings from this study will have implications for clinical practice, potentially guiding the development of targeted interventions to improve proprioception and optimize pain management and functional outcomes in SAIS patients. Therefore, the primary objectives of this cross-sectional study were twofold. Firstly, we aimed to evaluate and compare shoulder proprioception between SAIS and asymptomatic individuals using a digital inclinometer. This assessment would provide valuable insights into the proprioceptive deficits associated with SAIS. Secondly, we sought to examine the relationship between shoulder proprioception and two important clinical parameters: pain intensity and functional disability. We propose the following hypotheses: (1) Individuals with SAIS will exhibit impaired shoulder proprioception in the affected limb compared to the unaffected upper extremity. (2) There will be a positive correlation between impaired shoulder proprioception and higher pain intensity reported on the VAS and increased functional disability reported on the Shoulder Pain and Disability Index (SPADI) in individuals with SAIS.

## 2. Materials and Methods

### 2.1. Study Design

This comparative cross-sectional study was conducted from March 2020 to March 2023 at medical rehabilitation clinics affiliated with the University Hospital, where individuals with subacromial impingement syndrome were diagnosed and referred by an orthopedic physician to physical therapy.

### 2.2. Inclusion and Exclusion Criteria

#### 2.2.1. Subacromial Impingement Syndrome Group

Forty-two participants diagnosed with subacromial impingement syndrome were recruited for this study. The following inclusion criteria served as the rationale for selecting the participants: clinically diagnosed with subacromial impingement syndrome between 20 and 50 years of age; unilateral shoulder pain that is exacerbated at the end of the range of motion (ROM) and during extended periods of abduction without significant hypomobility, presenting a positive sign in the Hawkins–Kennedy test as well as the Neer sign simultaneously [20]. Participants were excluded if they had ≥50% limitation of active or passive shoulder ROM, a history of fracture involving the upper extremity, shoulder surgery, a full-thickness rotator cuff tear, shoulder instability, a history of shoulder surgery, systemic musculoskeletal disease, or shoulder pain with cervical spine motion, neurological disorders affecting proprioception, and any other musculoskeletal or systemic condition affecting shoulder function.

#### 2.2.2. Asymptomatic Group

For the control group, a total of 42 individuals without shoulder pain who had not received any shoulder treatment in the preceding 12 weeks were selected. Furthermore, they exhibited negative signs in the drop arm test, Hawkins–Kennedy test, and Neer sign. Controls were carefully matched to the study group based on age, handedness, and the affected shoulder.

### 2.3. Ethics

The Institutional Review Board at King Khalid University (REC# 2020-19-34) granted the study’s permission from an ethical standpoint before it could proceed. Before taking part in the study, each participant signed a document indicating that they had read and understood the information offered. During every stage of the research process, every effort was made to safeguard the participants’ right to privacy and retain their anonymity. The research was carried out in accordance with the principles that are defined in the Declaration of Helsinki. This ensured that the participants were treated ethically and that their rights were protected during the entirety of the research process.

### 2.4. Outcome Measures

During the initial screening, participants underwent a thorough evaluation conducted by a physiotherapist (examiner 1) during their initial physical therapy session. The evaluation encompassed a questionnaire that gathered sociodemographic details, pain characteristics, and a physical examination. Pain intensity and shoulder disability were assessed using self-reported questionnaires, namely the VAS and the SPADI. A different physiotherapist (examiner 2) conducted the proprioceptive assessment to ensure objectivity, remaining unaware of the participant’s group allocation. The study employed two primary outcome measures: shoulder proprioception and pain intensity.

#### 2.4.1. Shoulder Proprioception:

**Figure 1:**
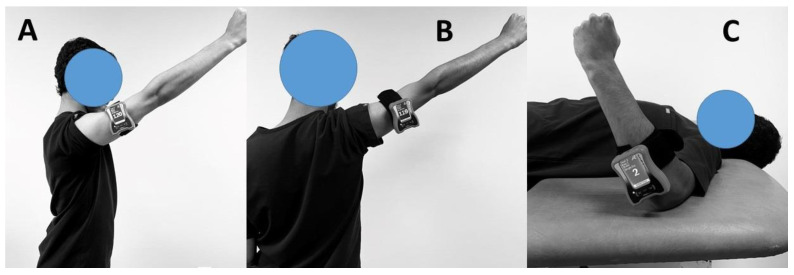
Assessment of shoulder proprioception in (**A**) Flexion; (**B**) abduction; and (**C**) medial and lateral rotations.

In order to assess the proprioceptive capabilities of the shoulder joint in the directions of flexion, abduction, medial rotation, and lateral rotation, a digital inclinometer was utilized (Figure 1).

The evaluation targeted proprioception during shoulder joint movements, including flexion, abduction at 60 and 120 degrees, and medial and lateral rotations at 10 and 20 degrees. The assessment was carried out using the Active Re-position Test, which involved three repetitions at each angle. During the evaluation, the participants were seated on armrest-free chairs with their feet fully in contact with the ground. The athletes’ knees were flexed at a 90-degree angle. To eliminate visual input, the athletes’ were blindfolded. A digital inclinometer was positioned where the deltoid muscle descends to insert into the humerus. A physiotherapist performed the test by moving the participant’s arms from the starting position (0 degrees) to the target angles (10, 20, 60, and 120 degrees). The arm remained at each target angle for 5 s, allowing the subjects to memorize the position before returning to the starting position. Subsequently, the subjects were instructed to independently move their arms towards each target angle. The difference between the target angle and the angle actively achieved by the athletes was measured and recorded in degrees. This process was repeated three times for each angle. The average difference obtained from these three tests at each angle was calculated and recorded. These recorded results were later used for statistical analysis.

#### 2.4.2. Pain Intensity

The VAS is a widely used subjective assessment tool for measuring pain intensity. It is a horizontal or vertical line, typically 10 cm in length, with verbal anchors at each end representing extremes of pain intensity, such as “no pain” and “worst imaginable pain [21]”. The participant is asked to mark a point on the line corresponding to their perceived pain intensity, with the distance from the “no pain” end serving as a quantitative measure of pain intensity. The VAS provides a continuous scale that allows individuals to express their pain experience along a continuum rather than being limited to predefined categories [22]. It is a reliable and valid method for pain assessment, offering a simple and intuitive way to capture pain intensity, monitor changes over time, and evaluate the effectiveness of pain management interventions [23]. The VAS is widely employed in clinical and research settings due to its ease of use, quick administration, and sensitivity in detecting subtle changes in pain intensity [23]. Pain intensity scores were recorded for each participant with SAIS.

#### 2.4.3. Shoulder Pain and Disability Index

The SPADI is a self-report questionnaire designed to assess the severity of shoulder pain and its impact on functional abilities and daily activities [24]. It comprises two subscales: “Pain” and “Disability”. The “Pain” subscale assesses the intensity and frequency of shoulder pain. In contrast, the “Disability” subscale evaluates the impact of pain on various functional tasks, such as self-care, work, and recreational activities [24]. Each subscale consists of multiple items rated on a Likert scale, where respondents indicate pain or difficulty experienced. After that, the results from each subscale are added together to get an overall SPADI score. This score can range from 0 to 100, with higher scores indicating greater levels of pain and impairment [24]. The SPADI enables clinicians and researchers to quantify and monitor changes in pain and disability over time, evaluate treatment outcomes, and tailor interventions to address specific areas of impairment [25].

### 2.5. Sample Size Estimation

A recent systematic review was used as a source for the standardized mean difference (SMD) used in our study’s determination of the appropriate sample size [9]. Among the proprioceptive variables analyzed, joint position sense exhibited statistically significant differences (SMD 1.19, 95% confidence interval [CI] 0.71–2.63, *p* < 0.001) in patients with SAIS. The meta-analysis used SMD as a summary statistic to standardize the results across multiple studies [9]. We adopted a conservative approach by selecting the lower confidence interval limit (0.94) as the effect size (Cohen’s d) between the case and control groups. With a significance level of 5% and a power of 95%, we performed a sample size calculation using a two-sided t-test for two independent samples. G*Power Software version 3.1.9 was used for the calculation. Considering the potential loss of participants during the study, a total of 42 participants were included in the analysis.

### 2.6. Statistical Analysis

The normal distribution of the study data was confirmed using the Shapiro–Wilk test. Descriptive statistics were used to summarize the demographic characteristics of the participants. The mean and standard deviation were reported for continuous variables, while frequencies and percentages were presented for categorical variables. An independent *t*-test was used to compare shoulder proprioception between the SAIS and the asymptomatic groups, and a correlation analysis (Pearson correlation coefficient) was performed to assess the relationship between shoulder proprioception, pain intensity, and functional disability. Statistical significance was set at *p* < 0.05. All analyses were performed using SPSS software (IBM, Version 22).

## 3. Results

This cross-sectional study comprised 42 individuals with SAIS and an equal number of asymptomatic participants, predominantly male. Table 1 presents the physical and demographic characteristics of the study participants, comparing the SAIS group with the asymptomatic group.

No significant differences were observed between the groups in age, gender, height, weight, or BMI. The SAIS group reported a pain intensity of 4.8 on the VAS, while the asymptomatic group did not report any pain. The SPADI scores, which measure shoulder disability, were only available for the SAIS group and showed percentages of 48.6 for pain, 29.7 for disability, and 36.3 for the total score.

Table 2 compares shoulder proprioceptive results between individuals with subacromial impingement syndrome (SAIS) and asymptomatic controls.

The SAIS group (*n* = 42) exhibited significantly higher joint position error (JPE) values compared to the asymptomatic group (*n* = 42) across all measurements. The mean differences (MD) between the groups were as follows: 3.00° (95% CI: 2.86, 3.64) for JPE in 60° of flexion; 3.25° (95% CI: 2.12, 2.74) for JPE in 120° of flexion; 2.43° (95% CI: 2.64, 3.36) for JPE in 60° of abduction; 2.26° (95% CI: 2.86, 3.64) for JPE in 120° of abduction; 3.54° (95% CI: 2.12, 2.74) for JPE in 10° of lateral rotation; 3.12° (95% CI: 1.97, 2.25) for JPE in 20° of lateral rotation; 3.24° (95% CI: 2.64, 3.36) for JPE in 10° of medial rotation; and 3.32° (95% CI: 2.86, 3.64) for JPE in 20° of medial rotation. All *p*-values were < 0.001. These results indicate that individuals with SAIS exhibit impaired proprioception compared to asymptomatic individuals.

**Figure 2:**
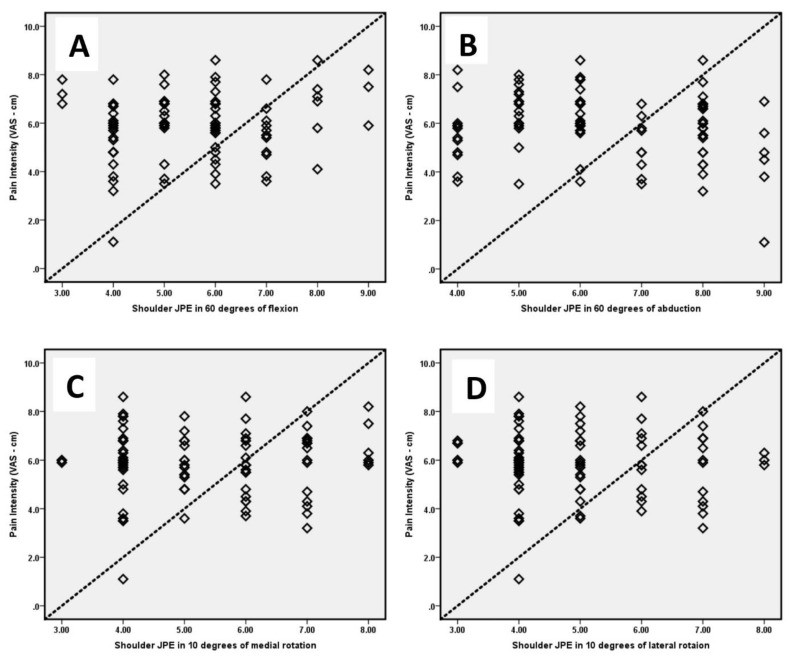
Correlation between pain intensity and shoulder joint position errors (JPE) in (**A**) 60° of flexion; (**B**) 60° of abduction; (**C**) 10° of medial rotation; and (**D**) 10° of lateral rotation.

Table 3 presents the correlation coefficients between shoulder joint position errors and patient self-reported measures of pain intensity (VAS) (Figure 2) and disability (SPADI).

Strong positive correlations were found between JPE at various angles of flexion and rotation and between pain intensity and disability. The correlation coefficients were as follows: for pain intensity (VAS), ranging from 0.61 to 0.71 (all significant at *p* < 0.01); and for disability (SPADI), ranging from 0.56 to 0.68 (all significant at *p* < 0.01). These results indicate that individuals with subacromial impingement syndrome (SAIS) and impaired shoulder proprioception tend to experience higher levels of pain intensity and disability, as reported on VAS and SPADI, respectively.

## 4. Discussion

The present study aimed to evaluate shoulder proprioception and its relationship with pain intensity and functional disability in individuals with SAIS. Our results revealed several important findings that contribute to our understanding of the impact of SAIS on proprioceptive function and its clinical implications.

To prevent causing discomfort to patients, we opted for shoulder testing movements at intermediate angles. It is already established that pain can affect proprioception accuracy in shoulder injuries. Therefore, we focused on evaluating the proprioceptive information obtained from the shoulder mechanoreceptors of individuals with SAIS and the influence of pain [26]. As a consequence, this study’s findings suggest that SAIS may include either abnormalities in the neurological pathway or damage to the mechanoreceptors [27]. Pain is likely to play a significant role in impairing proprioception in patients with SAPS. Therefore, it is crucial for clinicians to be mindful of the presence of pain symptoms when prescribing exercises, as they may potentially interfere with proprioceptive acuity in individuals with SAPS.

Regarding proprioception, our findings demonstrated that individuals with SAIS exhibited significantly higher JPE values compared to the asymptomatic group across all measurements. This suggests impaired shoulder proprioception in SAIS patients, supporting previous studies that have reported proprioceptive deficits in individuals with shoulder pathologies [28,29]. The impaired proprioception observed in our study could be attributed to the altered sensory input resulting from the anatomical changes and inflammatory processes associated with SAIS [13,30]. Proprioception relies on the integration of sensory input from mechanoreceptors within the joint, muscles, and tendons, which provide information about joint position and movement [31]. In SAIS, the anatomical changes and inflammatory processes associated with the condition can disrupt the normal sensory input and processing mechanisms, leading to impaired proprioception [32]. The altered sensory input may result from structural abnormalities, such as rotator cuff tendon inflammation, subacromial bursitis, or anatomical variations, impacting the transmission and interpretation of proprioceptive signals [27]. Furthermore, the inflammatory processes associated with SAIS can affect the sensory receptors within the joint, leading to altered proprioceptive feedback [27]. The impaired shoulder proprioception observed in our study highlights the importance of assessing proprioceptive function in SAIS patients. Proprioceptive deficits can have significant implications for motor control, joint stability, and functional performance. Individuals with impaired proprioception may exhibit altered muscle activation patterns, compromised joint coordination, and an increased risk of recurrent injuries. Therefore, it is crucial to address proprioceptive deficits in the management of SAIS to optimize treatment outcomes and enhance patients’ ability to perform daily activities.

The results of this study align with previous research conducted on the relationship between shoulder proprioception and pain intensity in individuals with subacromial impingement syndrome (SAIS). Several studies have reported strong positive correlations between joint position error (JPE) at various angles of flexion and rotation and pain intensity and disability. Similarly, Smith et al. [33] conducted a systematic review and reported a significant positive correlation between shoulder JPE, pain intensity, and disability scores. Their findings supported the notion that proprioceptive deficits were associated with greater pain severity and functional limitations. Another study by Sahin et al. [13] examined proprioceptive impairments in individuals with SAIS and reported significant positive correlations between shoulder proprioception deficits and pain intensity. Their findings indicated that individuals with higher pain levels tended to have greater proprioceptive deficits. Furthermore, a study by Atya et al. [34] investigated the relationship between proprioception and functional disability in patients with SAIS. They found a significant positive correlation between impaired proprioception and higher disability scores, suggesting that deficits in shoulder proprioception contributed to limitations in daily activities and impacted the quality of life of individuals with SAIS.

The consistent findings across these studies, including the current study, provide additional evidence for the clinical relevance of proprioceptive impairments in individuals with SAIS. The strong positive correlations between impaired shoulder proprioception and higher pain intensity and disability scores suggest that individuals with greater proprioceptive deficits tend to experience more severe pain and functional limitations [35,36,37]. The close relationship between proprioception and pain intensity may be attributed to the role of proprioceptive feedback in pain modulation [15,35,38]. Proprioceptive impairments can disrupt the normal neuromuscular control of the shoulder joint, leading to altered movement patterns and potentially contributing to pain amplification [39]. Additionally, the association between proprioception and functional disability highlights the impact of impaired proprioception on the ability to perform daily activities, which significantly affects the quality of life of individuals with SAIS [40].

The clinical significance of our study lies in highlighting the importance of assessing shoulder proprioception in individuals with SAIS. Understanding proprioceptive deficits and their correlation with pain intensity and functional disability can guide the development of targeted rehabilitation interventions. Proprioceptive training and exercises focusing on enhancing joint position sense and motor control could potentially improve pain management, functional outcomes, and overall quality of life in SAIS patients. Incorporating proprioceptive training into rehabilitation programs for SAIS should be considered to optimize treatment outcomes and enhance patient-centered care.

While our study provides valuable insights into the relationship between shoulder proprioception, pain intensity, and functional disability in SAIS, several limitations should be acknowledged. Firstly, the cross-sectional design limits our ability to establish causality and temporal relationships. Future longitudinal studies are needed to examine changes in proprioception, pain intensity, and disability over time and assess the effectiveness of interventions targeting proprioceptive deficits. Secondly, the sample size was relatively small, which may restrict the generalizability of the findings. Further studies with larger sample sizes are warranted to validate our results and enhance statistical power. Additionally, our study included predominantly male participants, limiting the generalizability of the findings to the broader SAIS population. Future research should aim to include a more diverse sample to better understand the bigger potential gender differences in proprioceptive impairments and their clinical implications in SAIS.

Limited shoulder ROM significantly impacts pain and disability in individuals with SAIS [41]. Impingement of tendons against the acromion during certain movements leads to irritation, inflammation, and persistent pain [42]. Restricted ROM in shoulder abduction and flexion worsens impingement and increases pain [42]. Limited shoulder ROM also contributes to muscle imbalances and altered movement patterns, resulting in compensatory mechanisms, altered muscle recruitment, and changes in joint biomechanics [43]. These compensatory mechanisms can further impair function, weaken muscles, and increase instability, perpetuating the cycle of pain and disability [43,44,45,46,47,48,49]. Addressing limited shoulder ROM through targeted rehabilitation interventions such as stretching, mobilization, and strengthening exercises is crucial for managing pain, restoring normal joint mechanics, and improving functional outcomes in SAIS [50]. By enhancing shoulder mobility and function, clinicians can effectively alleviate pain, enhance functional abilities, and improve the overall quality of life of SAIS patients.

## 5. Conclusions

This study provides evidence of impaired shoulder proprioception in individuals with SAIS compared to asymptomatic controls. The significant positive correlations between impaired proprioception, pain intensity, and functional disability emphasize the clinical relevance of proprioceptive deficits in SAIS. These findings highlight the necessity of adding proprioceptive assessment and focused rehabilitation therapies into the management of SAIS. Future research should focus on longitudinal studies with larger and more diverse samples to further elucidate the underlying mechanisms, explore gender differences, and evaluate the effectiveness of proprioceptive interventions in improving outcomes for individuals with SAIS.

## Figures and Tables

**Table 1:** Physical and demographic characteristics of study participants.

Variables	SAIS (*n* = 42)	Asymptomatic (*n* = 42)	*p*-Value
Age, years	34.8 ± 5.8	33.6 ± 6.2	0.238
Gender, male:female	28:14	26:16	0.346
Height, meters	1.68 ± 0.09	1.73 ± 0.05	0.122
Weight, kg	71.24 ± 5.96	69.58 ± 5.24	0.330
BMI, kg/m^2^	23.50 ± 2.17	23.38 ± 1.94	0.310
Right-handedness, %	100	100	-
Pain intensity, VAS: 0–10 cm	4.8 ± 2.1	-	-
Shoulder disability (SPADI)			
Pain %	48.6 ± 11.32	-	-
Disability %	29.7 ± 8.7	-	
Total %	36.3 ± 11.1	-	

BMI = body mass index; VAS = visual analogue scale; SPADI = shoulder pain and disability index.

**Table 2:** Comparison of shoulder proprioceptive results between SAIS and asymptomatic patients.

Variables	SAIS (*n* = 42)	Asymptomatic (*n* = 42)	MD	95% CI		*p*-Value
				Lower	Upper	
JPE in 60° of flexion (°)	3.23 ± 1.61	1.03 ± 1.12	3.00	2.86	3.64	<0.001
JPE in 120° of flexion (°)	3.86 ± 1.43	1.34 ± 0.78	3.25	2.12	2.74	<0.001
JPE in 60° of abduction (°)	3.31 ± 1.12	1.17 ± 0.35	2.43	2.64	3.36	<0.001
JPE in 120° of abduction (°)	3.93 ± 1.06	1.23 ± 0.42	2.26	2.86	3.64	<0.001
JPE in 10° of medial rotation (°)	3.43 ± 1.09	1.32 ± 0.94	3.54	2.12	2.74	<0.001
JPE in 20° of medial rotation (°)	3.86 ± 1.14	1.43 ± 0.82	3.12	1.97	2.25	<0.001
JPE in 10° of lateral rotation (°)	4.25 ± 1.34	1.07 ± 0.25	3.24	2.64	3.36	<0.001
JPE in 20° of lateral rotation (°)	3.94 ± 1.96	1.13 ± 0.32	3.32	2.86	3.64	<0.001

JPE = joint position error; SAIS = subacromial impingement syndrome; MD = mean difference; confidence interval.

**Table 3:** The correlation coefficients between shoulder proprioception and self-reported patient measures (VAS for pain intensity and SPADI for disability).

Variables		Pain Intensity (VAS)	SPADI		
			Pain	Disability	Total
JPE in 60° of flexion (°)	r	0.63 **	0.45 **	0.61 **	0.60 **
JPE in 120° of flexion (°)	r	0.62 **	0.56 **	0.59 **	0.62 **
JPE in 60° of abduction (°)	r	0.71 **	0.65 **	0.68 **	0.68 **
JPE in 120° of abduction (°)	r	0.70 **	0.67 **	0.64 **	0.64 **
JPE in 10° of medial rotation (°)	r	0.63 **	0.57 **	0.63 **	0.61 **
JPE in 20° of medial rotation (°)	r	0.71 **	0.58 **	0.60 **	0.62 **
JPE in 10° of lateral rotation (°)	r	0.63 **	0.49 **	0.62 **	0.60 **
JPE in 20° of lateral rotation (°)	r	0.61 **	0.56 **	0.60 **	0.59 **

JPE = joint position error; VAS = visual analogue scale; SPADI = shoulder pain and disability index; ** = correlation is significant at the 0.01 level (2-tailed).

## Data Availability

The corresponding author (R.S.R.) is prepared to promptly provide the requested data upon request, fostering transparency and facilitating scientific collaboration.
